# Clinical assessment of the TechArm system on visually impaired and blind children during uni- and multi-sensory perception tasks

**DOI:** 10.3389/fnins.2023.1158438

**Published:** 2023-06-02

**Authors:** Federica Morelli, Lucia Schiatti, Giulia Cappagli, Chiara Martolini, Monica Gori, Sabrina Signorini

**Affiliations:** ^1^Developmental Neuro-Ophthalmology Unit, IRCCS Mondino Foundation, Pavia, Italy; ^2^Department of Brain and Behavioral Sciences, University of Pavia, Pavia, Italy; ^3^Computer Science and Artificial Intelligence Lab and Center for Brains, Minds and Machines, Massachusetts Institute of Technology, Boston, MA, United States; ^4^Unit for Visually Impaired People, Istituto Italiano di Tecnologia, Genova, Italy

**Keywords:** assistive technologies, visual impairment, multisensory, rehabilitation, development

## Abstract

We developed the TechArm system as a novel technological tool intended for visual rehabilitation settings. The system is designed to provide a quantitative assessment of the stage of development of perceptual and functional skills that are normally vision-dependent, and to be integrated in customized training protocols. Indeed, the system can provide uni- and multisensory stimulation, allowing visually impaired people to train their capability of correctly interpreting non-visual cues from the environment. Importantly, the TechArm is suitable to be used by very young children, when the rehabilitative potential is maximal. In the present work, we validated the TechArm system on a pediatric population of low-vision, blind, and sighted children. In particular, four TechArm units were used to deliver uni- (audio or tactile) or multi-sensory stimulation (audio-tactile) on the participant's arm, and subject was asked to evaluate the number of active units. Results showed no significant difference among groups (normal or impaired vision). Overall, we observed the best performance in tactile condition, while auditory accuracy was around chance level. Also, we found that the audio-tactile condition is better than the audio condition alone, suggesting that multisensory stimulation is beneficial when perceptual accuracy and precision are low. Interestingly, we observed that for low-vision children the accuracy in audio condition improved proportionally to the severity of the visual impairment. Our findings confirmed the TechArm system's effectiveness in assessing perceptual competencies in sighted and visually impaired children, and its potential to be used to develop personalized rehabilitation programs for people with visual and sensory impairments.

## 1. Introduction

Since birth, humans rely on different sensory modalities to perceive the surrounding environment and they need to develop adaptive competencies to correctly interpret sensory information and act appropriately. Vision plays a crucial role in perceiving external information and developing a wide range of functional skills. For example, visual experience shapes the capability of orientation and localization in space and has an impact on the overall perceptual and cognitive development (Thinus-Blanc and Gaunet, 1997; Vasilyeva and Lourenco, 2010). Indeed, Bremner et al. (2011) demonstrated that visual information is predominant over vestibular information to guide orientation in both infants and toddlers. Also, vision guides the development of spatial skills, which are in turn crucial for social competencies (Cappagli and Gori, 2016). The central role of vision during the development is explained by its capability to simultaneously provide information about relationships between objects, individuals, and with the surrounding environment (Tinti et al., 2006; Bremner et al., 2011; Pasqualotto and Proulx, 2012). In support to this view, Cappagli et al. (2017a) found that also the perception of spatial information occurring through auditory and tactile modalities is affected by the presence of simultaneous visual information, suggesting that spatial information tends to be organized according to a visual frame of reference.

Investigating how spatial competencies develop when early visual experience is corrupted might further support this view. Over the last few decades, numerous studies have been conducted on visually impaired (VI) people to test the effect of visual deprivation on the overall development, especially in case of congenital visual impairments (e.g., in case of inherited retinal disorders). The classical assumption is that visual impairments promote a refinement of the residual senses, allowing for instance VI adults to perform equally or even better than sighted people in spatial tasks (compensatory hypothesis, see Collignon et al., 2009; Merabet and Pascual-Leone, 2010). Other evidences suggest that vision cannot be fully replaced by other sensory modalities in spatial tasks, causing for instance delays or deficits in the visually impaired population (general-loss hypothesis, see Eimer, 2004; Pasqualotto and Proulx, 2012). Gori et al. (2014) suggested that hearing and haptic spatial impairments in the blind might be explained in terms of missing cross-sensory calibration during the development, because vision (the optimal sensory modality for spatial tasks) cannot be used to calibrate the other senses during early stages of development. To date, only few studies investigated the relative impact of sensory modalities during early stages of development. For instance, Gori (2015) demonstrated that sighted children and adults rely more on visual than haptic information to solve size discrimination tasks, while younger children rely more on the haptic channel. Berto et al. (2021) recently hypothesized that interactions between audio and visual perception start only at a late stage of development, while in the very early stages of life auditory processing is independent of vision. At the same time, other studies support the hypothesis that early visual experience is essential to guide perception. For instance, Cappagli et al. (2017a) found that congenitally blind children are significantly impaired both in auditory distance discrimination and proprioceptive reproduction tasks. Moreover, extensive clinical research on VI children has shown the detrimental effects of early visual deprivation on navigation and spatial competencies (Giudice, 2018; Bathelt et al., 2019).

Overall, there is evidence that multisensory stimulation may be useful to prevent the negative impact of visual impairment on perceptual development. Indeed, results from experimental and clinical studies suggested that multisensory rehabilitation may provide long-term positive effects on the development of spatial cognition in VI children (Cappagli et al., 2017b, 2019; Cuturi et al., 2017; Morelli et al., 2020). This could be due to the positive effect of sensory redundancy on perception in ecological settings (Gori, 2015). For instance, it has been shown that multisensory stimulation improves the precision of perception and encoding of environmental events, with beneficial effects on the behavioral response to such events (Cao et al., 2019). These findings support the need for a deeper understanding about how the development and functioning of multisensory integration is affected by the absence of vision. Indeed, such a knowledge would provide a neuroscientific basis for the development of science-driven interventions in the context of rehabilitation, informing how to train residual perceptual competencies and elicit accurate responses. In this context, there is a strong need of developing novel technological tools, able to assess and train perceptual skills of VI people. This is especially valuable at an early age, when the brain plasticity is maximal and a proper training facilitates the emergence of adaptive skills. Indeed, the majority of technologies for VI people are developed for assistive purposes (e.g., Chebat et al., 2018), and they usually substitute vision by delivering information through the residual sensory modalities, rather than training such modalities and increase the subject's autonomy in retrieving useful information from the environment. However, recent works introduced the use of technological aids for rehabilitation purposes, and encouraging results were obtained both in adults (Cappagli et al., 2017b) and children (Cappagli et al., 2019).

Within this perspective, we developed the TechArm system, a technological tool designed to provide a quantitative assessment of different perceptual skills (residual vision, hearing, and touch) and to exploit unisensory and multisensory mechanisms in a rehabilitation setting. The system was designed to be easily operated by visually impaired patients from the very first years of life, and to be integrated by therapists and clinicians within their training protocols. The system proved its effectiveness in investigating perception on typical adults (Schiatti et al., 2020; Martolini et al., 2021). Here, the TechArm system was validated for the first time on visually impaired children, during uni- and multisensory perception tasks. Our purpose was to investigate the system's effectiveness in: (i) providing a quantitative measure of perceptive capabilities of visually impaired children (i.e., as an assessment tool); and (ii) providing sensory redundancy, which can potentially be used to train and improve the detection and discrimination of specific stimuli in the absence of vision, therefore yielding a rehabilitative function.

## 2. Materials and methods

### 2.1. Participants

Overall, 8 blind children (B, mean age 10.50 ± 4.21 y.o.), 23 children with low vision (LV, mean age 9.87 ± 3.09 y.o.), and 23 age-matched sighted children (S, mean age 9.83 ± 3.01 y.o.) participated in the study. Sighted children were recruited from primary and secondary schools in Genoa, Italy, while low-vision and blind children were recruited from the Neuro-Ophthalmology Unit at IRCCS Mondino, Pavia, Italy. The visual functions were assessed using a multiple optotype based on the age and level of instruction (Lea Vision tests or Snellen optotype, Hyvärinen et al., 1980). As inclusion criteria for VI children, in accordance with the International Statistical Classification of Diseases and Related Health Problems (World Health Organization, 2009) and the Italian Law 134/2001 defining visual impairments, we considered: (a) visual acuity between 1/10 and 3/10 (evaluated at 40 cm distance) for LV group, and residual vision from light/sporadic light to no light perception for B group (see Supplementary Table 1 for VI participants' clinical details); (b) a typical level of cognitive development (cutoff = 70), as assessed with the verbal scale of the Wechsler Intelligence Scale for Children (Grizzle, 2011); (c) visual deficit related to a peripheral (and not central) impairment (i.e., involving pre-geniculate structures). We did not included participants with: (a) a low-limit intellectual functioning (i.e., intellectual or development quotient < 70); (b) a diagnosis of auditory or neuromotor disorder besides the visual deficit.

### 2.2. Ethical approval

The local ethical committees (Comitato Etico della Regione Liguria and Comitato Etico Policlinico San Matteo Referente Area Pavia, for sighted and visually impaired groups, respectively) approved the study, and the participants' parents or their legal representatives were asked to sign written informed consent forms compliant to the Declaration of Helsinki, before participating in the experiment.

### 2.3. Setup and protocol

**Figure 1:**
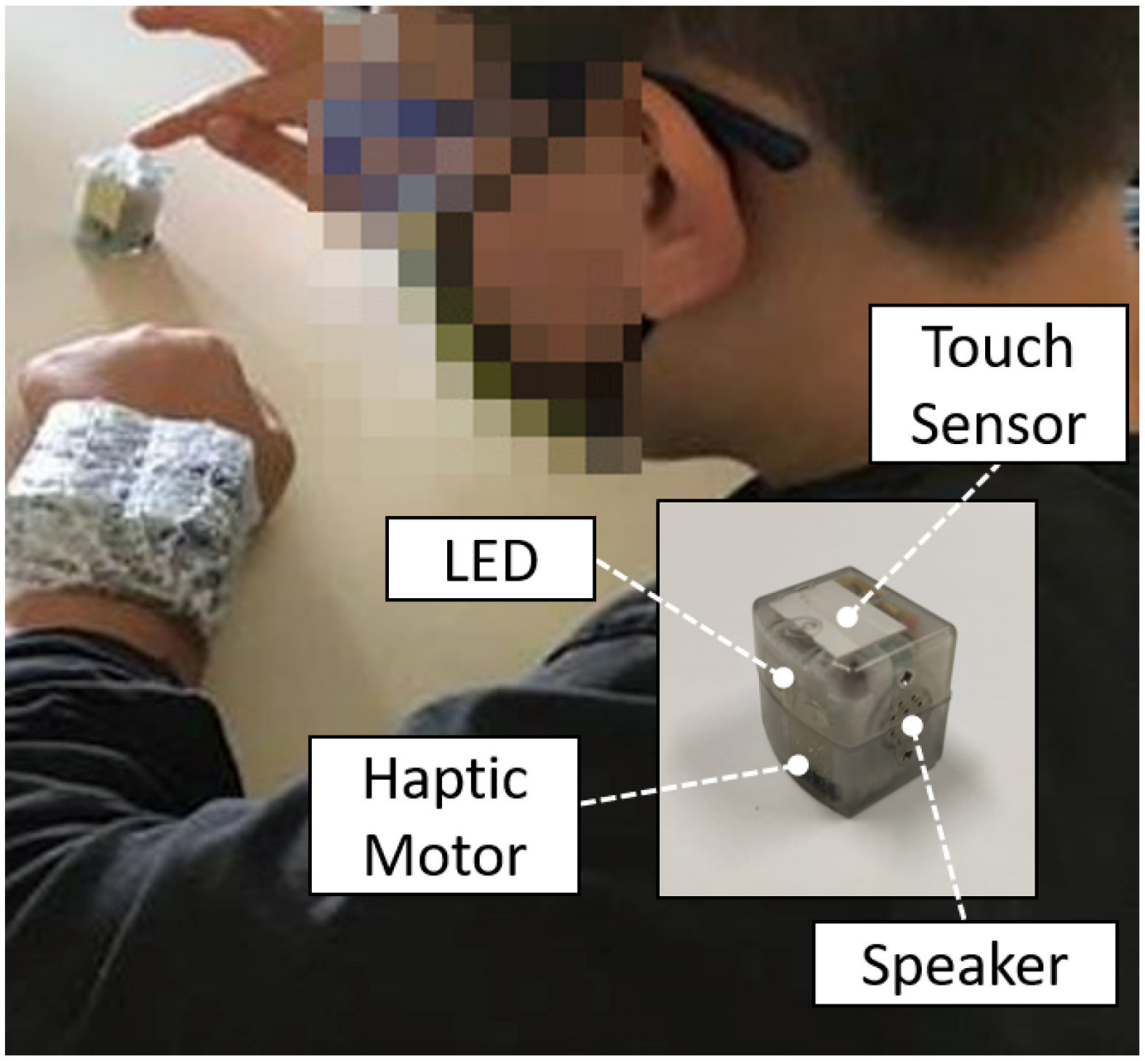
Experimental setup: four TechArm's units were placed on the subject's forearm and used to provide uni- and multi-sensory stimulation, i.e., audio, tactile, and audio-tactile stimuli, thanks to the embedded speaker and haptic motor. Visual stimulation (LED) was not used for this experiment. A fifth unit was used to stop the trial and collect the subject's response, by tapping on the touch sensor on the top of the unit.

The experimental setup consisted of the TechArm System (Figure 1), developed by the Electronic Design Laboratory and the Unit for Visually Impaired People at the Istituto Italiano di Tecnologia (Genoa, Italy) in collaboration with the Developmental Neuro-ophthalmology Unit (Pavia, Italy). It is a wearable, wireless system, composed by single cubic units (dimensions of a single unit: 2.5 × 2.5 × 2.5*cm*) able to provide spatially- and temporally-coherent multisensory stimulation, and to collect a real-time feedback from the user. In particular, each unit includes embedded actuators to enable visual (RGB light-emitting diode), auditory (digital amplifier and speaker), and tactile (haptic moto-driver) stimuli, and a capacitive sensor placed on the top surface of each unit to collect and record touch inputs from the user (dimension of the upper sensitized area: 6.25 *cm*^2^). An extensive technical description of the system is provided in Schiatti et al. (2020).

The experimental protocol was inspired by the one presented by Martolini et al. (2021). Four TechArm units were placed on the participant's left/right arm (right-handed/left-handed), with a 2 × 2 configuration, and used to deliver a uni- or multi-sensory stimulation to the subject during each trial. The dimension of the stimulation area was in the range of 6.25 *cm*^2^ (single unit) to 25 *cm*^2^ (four units). A fifth unit was placed on the table, next to the subject's right/left index finger, and it was used to collect the subject's response (Figure 1). Three stimuli conditions were implemented: audio (A), tactile (T), and audio-tactile (AT). Auditory stimuli were provided as a 79 dB white noise burst at 300 Hz, while tactile stimuli were conveyed by a vibro-motor (vibration frequency: 10 Hz). All stimuli lasted 100 ms, and 1 to 4 units were activated simultaneously during each trial, leading to 15 possible stimuli spatial configurations (4 for 1 active unit, 6 for 2 active units, 4 for 3 active unites, and 1 for 4 active units), and 45 trials in total (15 configurations × 3 sensory modalities), presented in randomized order. During each trial, the subject was instructed to tap the unit placed on the table as soon as possible after receiving the stimulation, regardless of the kind of stimulation conveyed, in order to stop the trial. Subsequently, the subject was asked to verbally report how many devices were perceived to be active (1, 2, 3, or 4). Five practice trials were allowed to make sure that subjects fully understood the task. The experiment lasted about half an hour, and short breaks were allowed at any time during the session.

### 2.4. Data analysis and statistics

We compared the performance of the three groups, by selecting a subset of 8 subjects from LV and S matching the average age of B (aged 10.63 ± 4.37 y.o. and 10.50 ± 4.21, respectively). Performance was assessed in terms of classification accuracy (ratio of correct answers over the total number of trials), considering as classes the number of active devices (1 to 4) for each sensory modality (A, T, and AT). Accuracy was computed from trials for each subject in each stimuli condition. Considering the fact that the classes are unbalanced, we also investigated the effect of two types of errors: (i) the detection of a certain class when the actual number of activated units belongs to one of the other classes (false positives, FPs), and (ii) the missed detection of a certain class when the actual number of active units belongs to it (false negatives, FNs). Specifically, we computed Precision (ratio of correctly classified trials, i.e., true positives, TP, over the sum of TP and FP), and Sensitivity (ratio of TP, over the sum of TP and FN) for each class and subject. The overall Precision and Sensitivity for each subject were computed by performing a weighted average of classes values, according to the total number of trials within each class. After verifying that the data did not follow a normal distribution (Shapiro-Wilk test), we performed a statistical analysis using non-parametric statistics. We ran three separate two-way permuted ANOVAs with Accuracy, Precision and Sensitivity as dependent variables respectively. We considered the group as between-factor (three levels: Blind—B, Low Vision—LV, and Sighted—S), and stimulation condition as within-factor (three levels: Auditory—A, Tactile—T, Audio-Tactile—AT). We performed *post-hoc* tests to assess the significant within- and between-group's differences. For both levels of analysis, the permuted Bonferroni correction for non-parametric data was applied in case of significant effects to adjust the *p*-value for multiple comparisons (significant value: α = 0.05). Considering the whole sample of LV, for each sensory condition, we computed the correlation (Pearson coefficient) among the individual performance (accuracy) and the degree of visual impairment, expressed in terms of Logarithm of the Minimum Angle of Resolution (LogMAR) at 40 cm (see Moussa et al., 2021 for the conversion between the near visual acuity expressed in decimal system and the logMAR chart).

## 3. Results

### 3.1. Performance analysis

**Figure 2:**
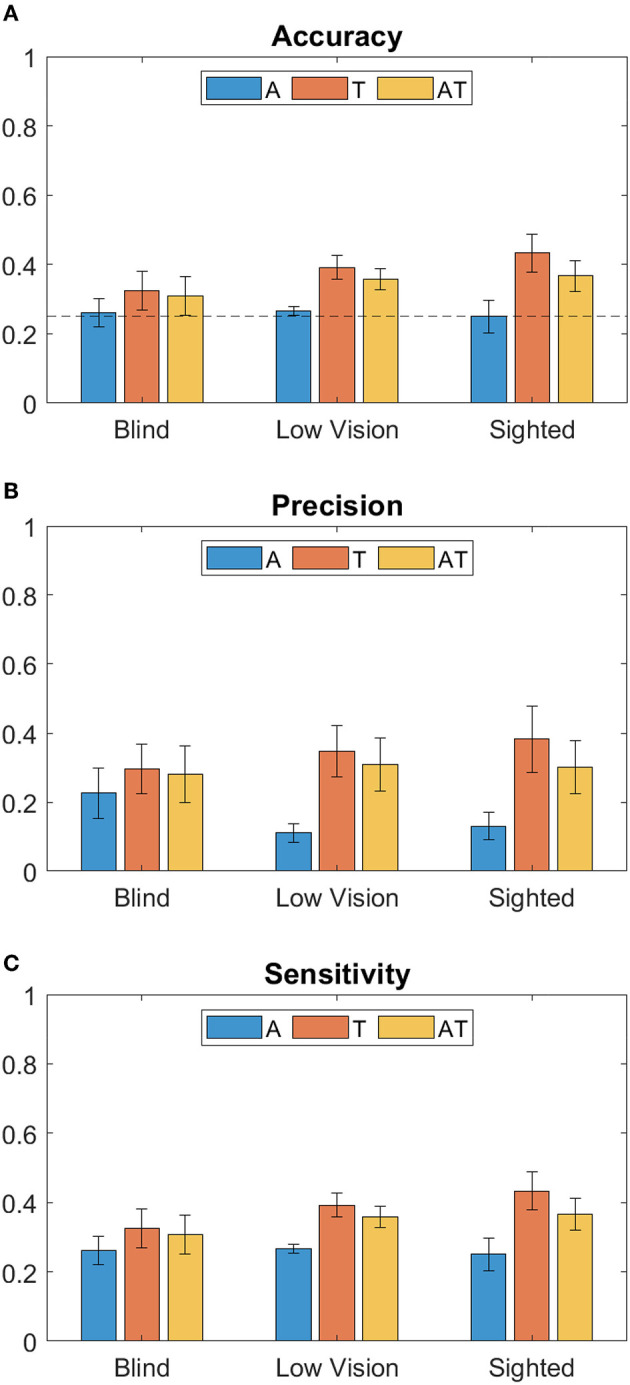
Bar plots of performance metrics (Mean ± Standard Error) for each group (Blind, Low Vision and Sighted), and different stimulation conditions (A, T, and AT, respectively, in blue, red and yellow color). From top to the bottom: **(A)** Classification Accuracy, with chance level = 0.25 indicated by a dotted line, **(B)** Precision and **(C)** Sensitivity.

**Figure 3:**
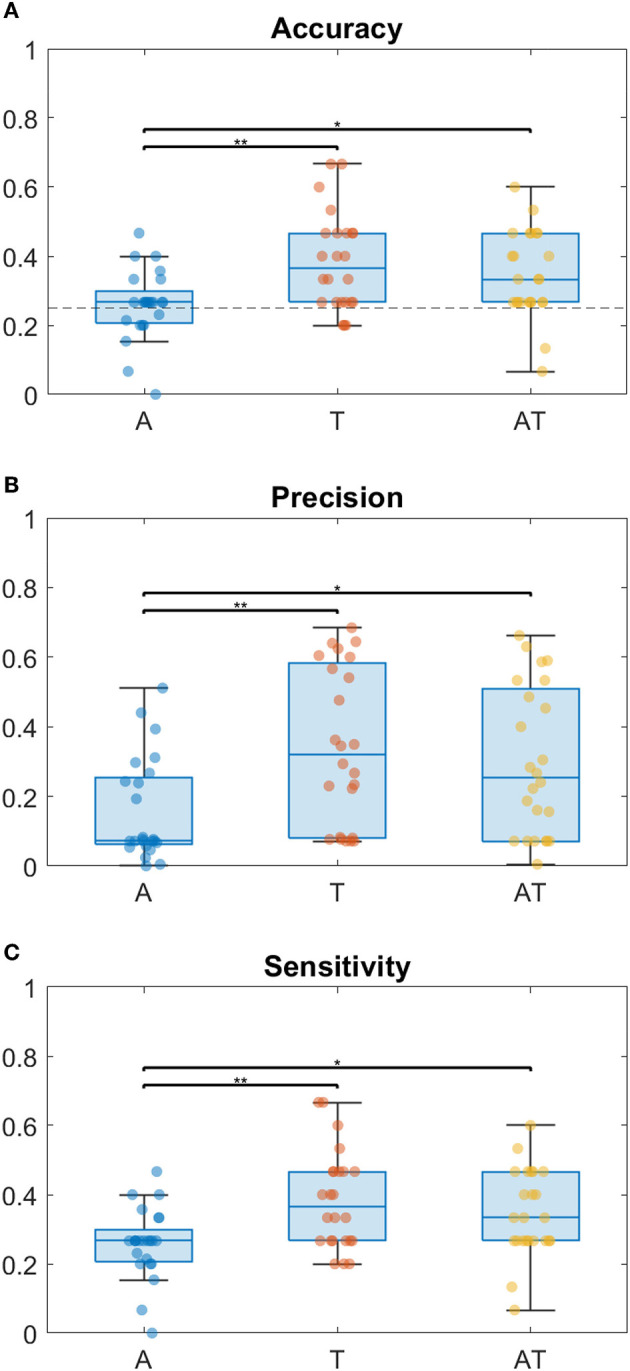
Box plots of performance metrics regardless of the group, with scatter plots of individual data, in different stimulation conditions (A, T, and AT, respectively, in blue, red and yellow color). From top to the bottom: **(A)** Classification Accuracy, with chance level = 0.25 indicated by a dotted line, **(B)** Precision and **(C)** Sensitivity. Horizontal bars on the top of the boxes report the statistical significance according to the non-parametric *post-hoc* tests (*p* < 0.01^**^ and *p* < 0.05^*^).

Considering the index of correct responses among the total of trials, results show that for all groups the best accuracy is achieved when using the T modality, followed by the AT (Figure 2A). While for T and AT, Sighted perform better than Low Vision and Blind children, the accuracy for A condition is around chance level for all groups. From the two-way ANOVA analysis considering Accuracy as dependent variable, the group (B, LV, S) and the stimuli condition (A, T and AT) as between- and within- factors respectively, we found a main effect only for the stimuli condition (Residual Sum of Squares *RSS* = 0.193, *iter* = 5, 000, *p* = 0.002). Subsequent *post-hoc* tests were therefore performed on data from different conditions, regardless of the group (as shown on the box plot in Figure 3A). They revealed no significant difference between T-AT, while accuracy in A condition is always worse than both in T (*p* < 0.01^**^) and AT (*p* < 0.05^*^) conditions. Figures 2B, C show results in terms of Precision and Sensitivity for each group and condition. Also the effect of false negative errors (correct number of active units not detected) is stronger for A than T and AT conditions, as evidenced by the lower Sensitivity values on Figure 2C. Interestingly, while both sighted and low vision children tend to be more sensitive than precise for A stimulation, Blind children show a similar level of Precision and Sensitivity when using the auditory modality. Again, from the ANOVA analysis we found a significant main effect of the stimuli condition for both Precision (*RSS* = 0.451, *iter* = 5, 000, *p* = 0.013) and Sensitivity (*RSS* = 0.194, *iter* = 5, 000, *p* = 0.012), and a null group's effect. By performing *post-hoc* tests on data from all groups, we found that both Precision and Sensitivity are significantly lower in A compared to T (*p* < 0.01^**^) and AT (*p* < 0.05^*^), as shown in Figures 3B, C.

### 3.2. Correlation analysis

**Figure 4:**
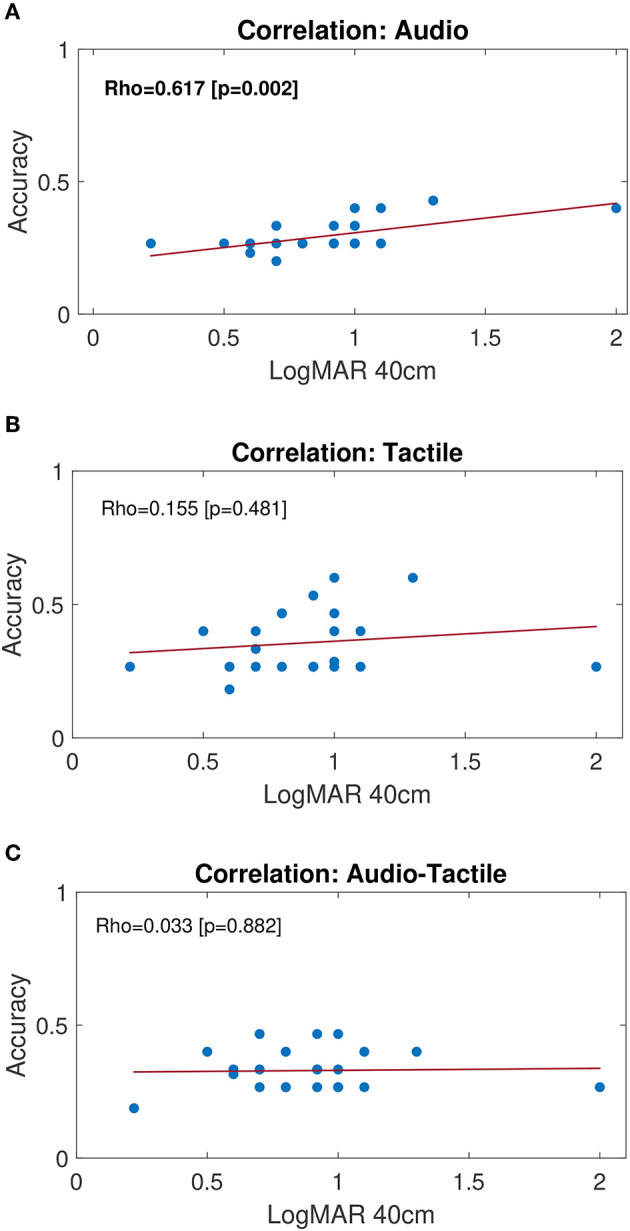
Correlation plots for LV group. Blue dots represent the scatter plot of individual performance (Accuracy) compared to residual vision (logMAR value for near vision, i.e., 40 cm). A higher LogMAR value corresponds to a lower visual acuity. Least-squared fit lines are shown in red. From top to the bottom, correlation plots refer to subjects' performance during: **(A)** Audio, **(B)** Tactile and **(C)** Audio-Tactile stimulation condition. Pearson correlation coefficient (ρ) and associated *p*-value are indicated within each plot. Bold text indicates significant correlation (*p* < 0.05).

Figure 4 shows the results of the correlation analysis performed on the full-size (*n* = 23) group of LV children. Specifically, the Pearson correlation coefficient was computed considering the distributions of Accuracy and the degree of visual impairment (LogMAR at 40 cm). Results show no significant correlation between the performance and the severity of the visual impairment for tactile and audio-tactile conditions, while a significant correlation (*p* = 0.002) was found for audio condition (Figure 4A). Children with a lower visual acuity (higher LogMAR values) show a higher capability of detecting differences in audio signals (higher Accuracy) than children retaining a higher level of residual vision (lower LogMAR values). Qualitatively, individual values on Figures 4B, C suggest that the performance in AT increases compared to A for LogMAR lower than 1, while it decreases for higher LogMAR values.

## 4. Discussion

It is well-known that congenital or early acquired visual impairments might interfere with developmental processes (Dale and Sonksen, 2002). Therefore, there is the need to design and clinically validate rehabilitation devices for VI children both to assess and train perceptual skills toward the interpretation of non-visual cues. The present study aimed at assessing the effectiveness of a novel technological tool (TechArm system) in a pediatric population of VI children.

First, our results suggest that the effect of sensory redundancy is the same across different visual conditions (visual impairment vs. normal vision). This result is in line with literature about multisensory development, e.g., audio-visual integration (Neil et al., 2006; Gori et al., 2014). Indeed, audio and visual information are typically integrated in the brain when originating from the same location, and this typically improves localization capabilities. However, such cross-modal interaction is not balanced among sensory modalities. Indeed, vision is predominant and it can bias auditory information (to an extent depending on conditions, such as the distance between the two different stimuli, see Körding et al., 2007 and Cao et al., 2019). Barutchu et al. (2010) observed that facilitation of motor responses to multimodal (audio-visual) stimuli is visible from the age of 6–7 years. At the same time, such response is inconsistent in older children, who show greater variability in responses compared to adults (Barutchu et al., 2010). It has been hypothesized that audio-visual integration may be task-dependent, therefore requiring the integrity and maturation of superior cognitive competencies, such as attention. For instance, a recent study suggested that multisensory gain in simple detection tasks may be related to a child's cognitive level (Barutchu et al., 2010; Denervaud et al., 2020). Also, it has been shown that audio-visual integration emerges later for spatial localization on a vertical plane compared to the horizontal plane, demonstrating visual dominance over audio for vertical multisensory perception (Gori et al., 2021). The majority of studies about perceptual abilities on multisensory integration involve vision, variously combined with hearing or touch, due to the vision's unique characteristic of conveying simultaneously a big amount of information about surrounding objects and about objects-events relationships. By contrast, sensory redundancy for non-visual stimuli (e.g., audio-tactile) has been less investigated, especially in children. Some researchers have hypothesized that the effectiveness of audio-tactile interactions in improving perceptual skills may depend on the spatial coincidence of the stimuli. Moreover, the impact of audio-tactile interactions seems to be affected by the type of the experimental task, e.g., detection vs. discrimination (Guest et al., 2002; Murray et al., 2005; for a review, see Kitagawa and Spence, 2006).

The present study demonstrated a predominance of touch over both only-auditory and multimodal audio-tactile stimuli during a perceptual task. The lower precision in A condition compared to T and AT suggests that subjects wrongly predict the number of active devices, by committing a false positive error, more frequently in A than in T and AT condition (Figure 2B). Such an effect of the sensory modality is significant regardless of the visual condition (i.e., it is evident both in VI and sighted children). In accordance with previous works, this suggests that the multisensory gain may be not only age-dependent but also task-dependent. Indeed, touch is the first sense to mature in typically developing infants (Smith, 1972) and it has a task-specific prevalence over the other sensory modalities. For example, touch is the most effective sense for size discrimination tasks in younger children, while vision provides more accurate responses in orientation tasks (Gori et al., 2008; for a review see Burr and Gori, 2012). Studies on VI people provided conflicting results about the performance of VI individuals (e.g., better performance in haptic recognition tasks, worse performance during tasks involving the recognition of spatial attributes of objects such as orientation, see Morrongiello et al., 1994; Alary et al., 2009). Our study suggests that in some cases multisensory integration may develop later and follow a different trajectory, both for VI and sighted children. In the absence of vision, touch proves to be the most effective sense in size discrimination tasks, enhancing perceptual precision over auditory-only, as evident from the better performance obtained for tactile stimuli than auditory stimuli. The dominance of touch over audition has been previously reported both for infants and adults in tasks of information processing in the peri-personal space, in connection with head and hand position (Sanabria et al., 2005; Thomas et al., 2018; Martolini et al., 2021). Furthermore, previous studies have shown that in audio-tactile interaction tasks, tactile stimuli are more important than auditory ones at the level of information processing (Hötting and Röder, 2004; Soto-Faraco et al., 2004). From these findings, we might speculate that hearing could be trained or calibrated by the haptic sense for this specific kind of task, in the lack of vision.

To assess whether different types of stimulation can affect performance in the group of LV children, we analyzed the correlation between the severity of visual impairment and performance during different stimuli conditions. Interestingly, we found an inverse correlation between the accuracy of auditory processing and the visual acuity, resulting in a better performance for children with severe visual impairments. The impact of visual deprivation on the development of other sensory modalities, such as hearing, has been previously demonstrated. Nevertheless, most of the existing studies were performed on blind people (see Gori et al., 2014 on a spatial localization task), and little is known about the extent to which the severity of the visual impairment may affect the development of hearing. In a recent study, Kolarik et al. (2020) showed that the auditory interpretation of distance and room size is related to the severity of a visual impairment, e.g., the higher the severity the larger both perceived distances and size of a room. Moreover, Senna et al. (2022) found that children with treated congenital cataract perform better than children with untreated cataract in an auditory bisection task, but worse compared to sighted controls, with a significant correlation between the performance and the post-surgical visual acuity. Such results suggest that an early sensory deprivation may alter the intact sensory modalities as well. Both behavioral, neuro-physiological and neuroimaging methods were used to investigate the differences in spatial hearing skills on adults with congenital, early-onset and late-onset visual impairments (Voss et al., 2004; Collignon et al., 2011, 2013; Tao et al., 2015). Results showed that in late-onset VI people the auditory spatial competencies seem to be affected by the duration of blindness, and the behavioral and cortical responses approach those of congenitally or early-blind people as blindness progresses over time. This highlights the important role of visual calibration during childhood, which decreases with prolonged blindness (Amadeo et al., 2019; Gori et al., 2020). To explain the better performance of blind children compared to low vision in audio modality, we hypothesize that children with mild visual impairments are more likely to rely on their residual vision in everyday life and during rehabilitation activities (Morelli et al., 2020), and therefore they perform similarly to sighted children. At the same time, they might not effectively use hearing when audio is the only modality provided. Anyhow, further data are needed to draw stronger conclusions. Indeed, the LV group we considered mainly presents mild low vision (see Figure 4). We could find a significant difference between LV and S by narrowing the inclusion criteria for the LV group, or by considering LV sub-groups with different degrees of impairment. Blind or severely impaired children, on the other hand, perform better on A condition because they likely rely on hearing as their most trained sense to compensate the lack of vision. Individual values on the plots also suggest that the performance in AT increases compared to A for LogMAR lower than 1, while it decreases for higher LogMAR values (see Figures 4B, C). Such observation discloses the TechArm system's effectiveness in shedding light on individual differences, and its potential as a tool to assess how perceptive mechanisms develop, while taking into account the subject-specific condition as well as the degree of visual impairment, and therefore allowing to plan customized rehabilitation protocols.

## 5. Conclusion

Here, we validated for the first time the TechArm system on a clinical population of blind and low-vision children, comparing results with a control group of sighted children. Our findings revealed the dominance of touch over hearing in sensory discrimination within the peripersonal space and a positive effect of sensory redundancy for stimuli localization. Furthermore, the inverse correlation between residual vision and auditory accuracy highlighted the importance of developing personalized training interventions. Indeed, a subject-specific assessment and rehabilitation program is essential to identify the most impaired functional competencies, such as orientation and mobility, and effectively improve the person's autonomy during everyday-life activities. Our system proved to be effective at different levels, e.g., by allowing to compare perception-related skills using different sensory modalities and to disentangle the contribution of each sensory channel during a multisensory perceptual task. Beyond the protocol implemented for this study, the system can facilitate the design of different task conditions aimed at investigating perceptual capabilities in uni- and multi-sensory tasks, by varying both the overall stimulation area and the number of simultaneously activated sensory modalities. For instance, the system would allow to perform both reaching-to-the-body and reaching-to-the-sound tasks in which children are respectively required to localize sensory stimulation on their own body (e.g., to increase body awareness) and localize stimulation in the near and far space (e.g., to increase mobility and orientation skills). Overall, these results support the TechArm's potential as a tool with a bi-fold function as: (i) an assessment device, to provide quantitative measures about the role played by different sensory modalities in perception, when a sensory deprivation occurs, and (ii) a rehabilitative device, which could be integrated within personalized training programs, according to each patient's sensory and cognitive profile, for the rehabilitation of impaired perceptual functions.

## Data availability statement

The raw data supporting the conclusions of this article are available at the doi 10.5281/zenodo.7651325, without undue reservation.

## Ethics statement

The studies involving human participants were reviewed and approved by Comitato Etico della Regione Liguria (Genova, Italy, prot. IIT_UVIP_COMP_2019 N. 02/2020) and Comitato Etico Policlinico San Matteo Referente Area Pavia (Pavia, Italy, prot. p-20190078848). Written informed consent to participate in this study was provided by the participants' legal guardian.

## Author contributions

FM and LS contributed to the study design and implementation, data collection, data analysis, and article writing and reviewing. GC contributed to the study design, data collection support, data analysis, and article writing and reviewing. CM contributed to the study design and data collection. SS and MG contributed to the study conception, supervision, funding, and article reviewing. All authors contributed to the article and approved the submitted version.
