# Supplementary material for: Clinical assessment of the TechArm system on visually impaired and blind children during uni- and multi-sensory perception tasks
Source: Front Neurosci. 2023 Jun 2;17:1158438. doi: 10.3389/fnins.2023.1158438 (PMC10272406; doi:10.3389/fnins.2023.1158438)
Supplement: Supplementary file 1 [file Data_Sheet_1.PDF]

frontiers

Supplementary Material

1 CLINICAL DETAILS OF VISUALLY IMPAIRED PARTICIPANTS

Table S1. Clinical details of visually impaired participants. From left to right: a) Visual Condition, either low vision (visual acuity lower than 3/10) or blind (light/sporadic light to no light perception, see Organization et al. (2009)); b) Age; c) Gender; d) Visual Acuity expressed in terms of LogMAR at a 3m distance; e) Visual Acuity expressed in terms of LogMAR at a 40cm distance; f) Clinical diagnosis of the visually impaired condition.

Visual condition	Age	Gender	Visual Acuity LogMAR (3 m distance)	Visual Acuity LogMAR (40 cm distance)	Diagnosis
low vision	8	M	0.8	0.6	inherited retinal dystrophy
low vision	8	M	1	0.8	inherited retinal dystrophy
low vision	8	M	0.8	1	inherited retinal dystrophy
low vision	16	F	0.4	0.5	inherited retinal dystrophy
low vision	7	M	1	0.7	eye maldevelopment
low vision	8	M	1	0.7	inherited retinal dystrophy
blind	7	F	no answers	light perception	inherited retinal dystrophy
low vision	9	M	no answers	2	eye maldevelopment
blind	9	M	no answers	no answers	anophthalmia
blind	7	M	no answers	light perception	inherited retinal dystrophy
low vision	12	M	1	0.22	congenital cataract
low vision	6	M	1	1	congenital cataract
low vision	17	M	1	1.1	optic nerve hypoplasia
low vision	8	F	1	0.6	congenital cataract
low vision	6	F	no answers	1	inherited retinal dystrophy
low vision	10	F	no answers	1.1	eye maldevelopment
low vision	9	F	1	0.92	congenital glaucoma
low vision	9	M	1	0.92	metylmalonic acidemia
low vision	9	M	0.8	0.7	inherited retinal dystrophy
low vision	7	M	1	0.92	inherited retinal dystrophy
low vision	11	M	0.8	1	optic nerve hypoplasia
low vision	13	F	no answers	1	retinopathy of prematurity
blind	9	F	no answers	no answers	anophthalmia
low vision	13	M	no answers	0.8	retinopathy of prematurity
low vision	12	M	no answers	1.3	inherited retinal dystrophy
low vision	6	F	1	0.92	eye maldevelopment
low vision	14	F	1	0.8	optic nerve hypoplasia
blind	6	M	no answers	light perception	retinopathy of prematurity
blind	17	F	no answers	no answers	inherited retinal dystrophy
blind	15	M	no answers	no answers	inherited retinal dystrophy
blind	14	M	no answers	no answers	retinopathy of prematurity

REFERENCES

Organization, W. H. et al. (2009). International statistical classification of diseases and related health problems

Table S2. Analysis of Variance Table for the full size B (n=8), LV (n=23) and S (n=23) samples of subjects. Results of the two-way ANOVA analysis are reported considering either Accuracy, Precision or Sensitivity as dependent variable, and the group (B, LV, S) and stimuli condition (A, T and AT) as between- and within- factors. Results of multiple comparisons are reported in terms of p-values from the post-hoc t-tests with Bonferroni correction for all stimuli conditions pairs: Audio vs Tactile (A-T), Audio vs Audio-Tactile (A-AT) and Tactile vs Audio-Tactile (T-AT). Significance codes: $p < 0.001$ '***', $p < 0.01$ '**', $p < 0.05$ '*'.

		Df	RSS	RMS	Iter	Pr(Prob)	Post-hoc t-tests (p)	
Accuracy	GROUP	2	0.022	0.011	470	0.302	6e-4***	A-T
	CONDITION	2	0.165	0.083	5000	0.005 **	4.8e-3***	A-AT
	GROUP:CONDITION	4	0.010	0.002	135	1.000	0.979	T-AT
	Residuals	108	1.497	0.014				
Precision	GROUP	2	0.022	0.012	51	1.000	0.001***	A-T
	CONDITION	2	0.378	0.189	5000	<2e-16 ***	0.016*	A-AT
	GROUP:CONDITION	4	0.068	0.017	56	0.982	1	T-AT
	Residuals	108	3.895	0.036				
Sensitivity	GROUP	2	0.022	0.011	212	0.401	<1e-16 ***	A-T
	CONDITION	2	0.165	0.083	5000	0.001 ***	0.002***	A-AT
	GROUP:CONDITION	4	0.010	0.002	92	0.989	0.912	T-AT
	Residuals	108	1.497	0.014				
